# Upper limb sensorimotor recovery in Asian stroke survivors: a study protocol for the development and implementation of a Technology-Assisted dIgitaL biOmaRker (TAILOR) platform

**DOI:** 10.3389/fneur.2023.1246888

**Published:** 2023-11-30

**Authors:** Hsiao-Ju Cheng, Lay Fong Chin, Christoph M. Kanzler, Rea Lehner, Christopher W. K. Kuah, Simone Kager, Eva Josse, Tengiz Samkharadze, Ananda Sidarta, Pablo Cruz Gonzalez, Eloise Lie, Monika Zbytniewska-Mégret, Seng Kwee Wee, Phyllis Liang, Roger Gassert, Karen Chua, Olivier Lambercy, Nicole Wenderoth

**Affiliations:** ^1^Singapore-ETH Centre, Future Health Technologies Programme, CREATE Campus, Singapore, Singapore; ^2^Institute of Rehabilitation Excellence (IREx), Tan Tock Seng Hospital Rehabilitation Centre, Singapore, Singapore; ^3^Rehabilitation Engineering Laboratory, Department of Health Sciences and Technology, ETH Zürich, Zürich, Switzerland; ^4^Rehabilitation Research Institute of Singapore, Nanyang Technological University, Singapore, Singapore; ^5^Singapore Institute of Technology (SIT), Singapore, Singapore; ^6^Lee Kong Chian School of Medicine, Nanyang Technological University, Singapore, Singapore; ^7^Neural Control of Movement Laboratory, Department of Health Sciences and Technology, ETH Zürich, Zürich, Switzerland

**Keywords:** stroke, neurorehabilitation, upper limb, assessment, recovery, sensorimotor impairments

## Abstract

**Background:**

Stroke is a leading cause of lifelong disability worldwide, partially driven by a reduced ability to use the upper limb in daily life causing increased dependence on caregivers. However, post-stroke functional impairments have only been investigated using limited clinical scores, during short-term longitudinal studies in relatively small patient cohorts. With the addition of technology-based assessments, we propose to complement clinical assessments with more sensitive and objective measures that could more holistically inform on upper limb impairment recovery after stroke, its impact on upper limb use in daily life, and on overall quality of life. This paper describes a pragmatic, longitudinal, observational study protocol aiming to gather a uniquely rich multimodal database to comprehensively describe the time course of upper limb recovery in a representative cohort of 400 Asian adults after stroke. Particularly, we will characterize the longitudinal relationship between upper limb recovery, common post-stroke impairments, functional independence and quality of life.

**Methods:**

Participants with stroke will be tested at up to eight time points, from within a month to 3 years post-stroke, to capture the influence of transitioning from hospital to community settings. We will perform a battery of established clinical assessments to describe the factors most likely to influence upper limb recovery. Further, we will gather digital health biomarkers from robotic or wearable sensing technology-assisted assessments to sensitively characterize motor and somatosensory impairments and upper limb use in daily life. We will also use both quantitative and qualitative measures to understand health-related quality of life. Lastly, we will describe neurophysiological motor status using transcranial magnetic stimulation.

**Statistics:**

Descriptive analyses will be first performed to understand post-stroke upper limb impairments and recovery at various time points. The relationships between digital biomarkers and various domains will be explored to inform key aspects of upper limb recovery and its dynamics using correlation matrices. Multiple statistical models will be constructed to characterize the time course of upper limb recovery post-stroke. Subgroups of stroke survivors exhibiting distinct recovery profiles will be identified.

**Conclusion:**

This is the first study complementing clinical assessments with technology-assisted digital biomarkers to investigate upper limb sensorimotor recovery in Asian stroke survivors. Overall, this study will yield a multimodal data set that longitudinally characterizes post-stroke upper limb recovery in functional impairments, daily-life upper limb use, and health-related quality of life in a large cohort of Asian stroke survivors. This data set generates valuable information on post-stroke upper limb recovery and potentially allows researchers to identify different recovery profiles of subgroups of Asian stroke survivors. This enables the comparisons between the characteristics and recovery profiles of stroke survivors in different regions. Thus, this study lays out the basis to identify early predictors for upper limb recovery, inform clinical decision-making in Asian stroke survivors and establish tailored therapy programs.

**Clinical trial registration:**

ClinicalTrials.gov, identifier: NCT05322837.

## 1 Introduction

At least 50% of stroke survivors suffer from upper limb impairments in the acute stage (1, 2), and the variety of recovery profiles widely vary (3, 4). Gaining a better understanding of the neurophysiological, behavioral and contextual factors affecting recovery post-stroke is essential to developing more tailored and optimized upper limb rehabilitation approaches and reducing the risk of persistent upper limb impairments (5, 6), which are known to lead to poor self-efficacy (7) and low self-reported health-related quality of life (Hr-QOL) (8, 9). However, this relies on rich data sets tracking upper limb sensorimotor recovery with sufficient time resolution and for a sufficiently long time beyond the initial hospitalization, and considering the multi-dimensional nature of upper limb deficits and their impact on quality of life.

During the last few years, understanding the time course of recovery after stroke as well as its major determinants has received increasing attention (10–14). However, only few data sets have collected information (i) with a high temporal resolution (i.e., multiple time points during both acute/subacute and chronic phases), (ii) over a time period longer than 1 year [but see (14, 15)], (iii) in a large and representative patient sample, and (iv) through the lens of multiple domains of functioning, Hr-QOL and disability. First available data sets, particularly measuring the recovery of upper limb function (3, 4, 16, 17), have revealed important new insights into predicting rehabilitation outcomes (3, 16) and, recently, also trajectories of recovery (4). Here, we aim to investigate the added value of technology-assisted assessments in an Asian cohort longitudinally. “Digital biomarkers,” i.e., metrics derived from technology-assisted assessments (18–20), offer the advantage of providing validated (21–23), objective and traceable descriptions of upper limb behavior on sensitive, continuous scales, often without ceiling effects (24). Digital biomarkers are helpful to complement clinical scores, uniquely inform on movement quality and allow for separating behavioral restitution from compensatory strategies (25), a key aspect that existing longitudinal studies on recovery failed to capture (10, 13).

To reach this aim, we designed a study protocol to gather a rich multimodal data set tracking the time course of upper limb recovery in a representative cohort of 400 Asian adults after ischemic or hemorrhagic stroke for up to 3 years and characterize the longitudinal relationship between upper limb recovery and independence/quality of life of post-stroke individuals. Compared to Caucasian stroke survivors, Asian stroke survivors have a higher incidence of intracerebral hemorrhages (ICH), lacunar ischemic strokes (IS) and strokes in the young (<45 years) as well as differential recovery patterns between ICH and IS strokes (26–28). However, there is scarce evidence on the profiles of the Asian stroke population. To answer this, we will employ a carefully selected battery of standardized clinical tests, technology-assisted assessments, neurophysiological assessments, tools for measuring Hr-QOL, as well as qualitative interviews with longitudinal follow-ups to uncover different recovery profiles in Asian stroke survivors. Our data set will, thus, assess multiple domains of upper limb recovery with a focus on sensorimotor function.

Such a longitudinal, comprehensive data set will yield clinically important information on key factors influencing post-stroke upper limb sensorimotor recovery trajectory, activity capacity, activity performance, real-world participation, and quality of life over time. We will use this data set to answer the following research questions: (i) Can digital biomarkers revealed by technology-based assessments and wearables deepen our understanding of post-stroke upper limb recovery when compared to neurophysiological measurements (transcranial magnetic stimulation, TMS), standardized clinical assessments, patient self-reports, and Hr-QOL? (ii) What is the interaction and interdependence between sensorimotor impairments, clinically assessed performance, neurophysiology, quality of life, and upper limb usage in daily life, for stroke survivors? (iii) What are the major determinants of recovery? This data set will provide a new means for quantifying the “natural course” of post-stroke recovery in accordance with the standard of care, providing a strong data-driven foundation on which future therapy could be potentially optimized.

## 2 Methods and analysis

### 2.1 Study design and participants

This study is a single-center, prospective longitudinal, observational cohort study. The study was registered on ClinicalTrials.gov with the following registration number: NCT05322837.

In this study, we aim to recruit up to 400 stroke survivors from the Tan Tock Seng Hospital (TTSH) Rehabilitation Centre inpatient stroke rehabilitation unit. TTSH Rehabilitation Centre is the largest stroke rehabilitation hospital in Singapore with a catchment area covering roughly 1/3 of the country. It provides customized stroke upper limb management which includes one-to-one individualized training, upper limb group intensive training, self-directed upper limb therapy, robotic upper limb retraining and neuromuscular electrical stimulation. Our sample of stroke survivors and can be deemed unselected. The inclusion criteria are as follows.

- Stroke confirmed by neurologists, neurosurgeons, and brain imaging (computed tomography (CT), computed tomography angiogram, magnetic resonance imaging (MRI), magnetic resonance angiogram).
- Asian ethnicity.
- Age 21–90 years.
- Montreal Cognitive Assessment (MoCA) scores 18/30 and above.
- Admission to the inpatient stroke rehabilitation unit within 8 weeks of stroke onset.
- Admission Fugl–Meyer score <66.

For qualitative interviews at >6 months post-stroke:

- Capacity to communicate.
- MOCA >24/30 at 6 months.

The exclusion criteria are below.

- Recurrent stroke with modified Rankin score (mRS) of >2 (i.e., recurrent stroke with mRS 0, 1, 2 can be included).
- Upper limb impairment related to conditions of subarachnoid hemorrhage, traumatic brain injury or brain tumors.
- Bilateral strokes leading to upper limb impairments.
- Uncontrolled medical conditions such as hypertension, hypotension, diabetes mellitus, unstable angina, cardiac failure, or sepsis will be excluded.
- Fractures or arthritis of upper limb joints/bones in the affected upper limb.
- Visual Analogue Scale (VAS) pain >5/10 in the affected upper limb.
- MoCA <18/30.
- Severe behavioral disturbance or agitation or epilepsy or other contradictions preventing the study participation.
- Life expectancy <6 months.
- End organ failures on replacements (renal dialysis or renal replacement therapies).
- Minimally responsive or unresponsive awareness (vegetative) states.
- Pregnancy or lactation states.
- Admission to the inpatient stroke rehabilitation unit later than 8 weeks post-stroke.
- (Related to TMS assessment only) a history of epilepsy or seizures, cranial surgeries, metal implants in the body or head, implanted electronics, metallic valves, skull fracture or brain injury, or head or brain surgeries.

For qualitative interviews only at >6 months post-stroke:

- Inability to communicate (aphasia, apraxia and dysarthria).

All participants will undergo their routine care in the inpatient stroke rehabilitation unit. Participation in this study will not have an impact on their intervention plans over time. Depending on their conditions at the discharge time point, they will be discharged to discontinue rehabilitation or to continue their rehabilitation in a community hospital, nursing home, other facilities such as specialist rehabilitation clinics, day rehabilitation centers or at home. After they are discharged, they will be invited back to the same hospital but at a different study site—TTSH Clinic for Advanced Rehabilitation Therapeutics (CART) for follow-up assessments. All therapists in the TTSH Rehabilitation Centre inpatient stroke rehabilitation unit and CART were jointly trained for all clinical and technology-assisted assessments to ensure good reliability.

To better capture the time course of post-stroke upper limb recovery, we aim to conduct clinical, neurophysiological, Hr-QOL, and technology-assisted assessments at various time points arching early subacute, late subacute, and chronic stages following stroke. To further understand the environmental and personal factors in community-dwelling stroke survivors, qualitative data on Hr-QOL related specifically to upper limb recovery in the chronic stages of stroke will also be collected in a subset of participants who have sufficient cognitive function and are willing to share their experience after stroke.

The fastest rate of neurological and functional recovery typically occurs during the first four to 6 weeks following the onset of stroke. As such, we will assess stroke survivors regularly from recruitment to discharge. To establish a baseline, we will collect comprehensive data covering motor, somatosensory and cognitive functions using standardized clinical, neurophysiological, and technology-assisted assessments as soon as patients are admitted to the inpatient stroke rehabilitation unit and recruited in our study (T0). This typically occurs 2–4 weeks post-stroke, as patients are initially treated in the acute unit of the hospital where we unfortunately are not able to perform any assessments. We then follow up on gains in motor and somatosensory function on a fortnightly basis (T1, 4–6 weeks post stroke). Close to expected discharge (T2, typically within 6–8 weeks post stroke), we will again assess motor and somatosensory functions using standardized clinical and technology-assisted assessments. If a participant is admitted later than 2–4 weeks (but no more than 8 weeks) post-stroke, recruitment in the study will still be considered, and T0 will be administered. Following discharge, the time points commonly reported in the literature of 3, 6, and 12 months post-stroke are selected, in addition to further follow-up assessments at 2 and 3 years post-stroke. Considering the change of living environment from bedside to the community, besides routine assessments on the motor, somatosensory, and cognitive functions, we additionally assess Hr-QOL to understand the impact of stroke-induced deficits on daily life. The overview of the assessment time points and assessment tools is described in Table 1.

**Table 1:** Overview of the assessment time points and assessment tools.

Time point		T0^a^	T1^a^	T2^a^	T3	T4	T5	T6	T7
Time post-stroke		2–4 weeks	4–6 weeks	6–8 weeks	3 months	6 months	1 year	2 years	3 years
Description	Assessment tool								
Global disability level	Modified Rankin Scale (mRS)					×	×	×	
Upper limb motor impairments	Fugl-Meyer Assessment of the Upper Extremity (FMA-UE)	×	×	×	×	×	×	×	×
	Shoulder Abduction and Finger Extension (SAFE) score	×							
	Grip strength	×	×	×	×	×	×	×	×
Upper limb activity capacity	Action Research Arm Test (ARAT)	×	×	×	×	×	×	×	×
Upper limb somatosensory impairments	Erasmus version of the Nottingham Sensory Assessment (NSA)	×	×	×	×	×	×	×	×
Upper limb spasticity	Modified Ashworth Scale (MAS)	×		×	×	×	×	×	×
Upper limb pain	Visual Analogue Scale (VAS)	×	×	×	×	×	×	×	×
Confidence in upper limb use	Upper Limb Self-Efficacy Test (UPSET)	×		×	×	×	×	×	×
Neglect	Bells Test	×							
Trunk impairment	Trunk Impairment Scale (TIS)	×							
Functional Independence	Functional Independence Measure (FIM)	×		×					
Cognitive impairments	Montreal Cognitive Assessment (MoCA)	×				×	×	×	×
Quality of life	EQ-5D			×	×	×	×	×	×
	Stroke Specific Quality of Life (SSQOL)				×	×	×	×	×
Neurophysiology^b^	Motor Evoked Potentials (MEPs)	×							
Technology-assisted assessment	Virtual Peg Insertion Test (VPIT)	×		×	×	×	×	×	×
	ETH Motor Impairment and Kinesthetic Evaluation (MIKE)	×		×	×	×	×	×	×
	ZurichMove	×		×	×	×	×	×	×
Psychosocial questionnaires^c^						×	×	×	
Qualitative interviews^c^						×	×	×	

a Time post-stroke may vary depending on the time admitted to the TTSH Rehabilitation Centre inpatient stroke rehabilitation unit. T1 will be 2 weeks after T0 and T2 will be 2 weeks after T1.

b Neurophysiological assessment will only be conducted in participants with SAFE score <5.

c Psychosocial questionnaires and qualitative interviews will be conducted in selected participants with sufficient cognitive function and willingness to share experiences coping with stroke.

As the nature of this study is explorative and as there is no generally accepted approach to estimate the sample size requirements for multivariate regression models (29), a classical sample size estimation was not possible. Instead, our target sample was based on previous studies aiming at understanding post-stroke upper limb recovery and logistical considerations. Existing studies have typically reported longitudinal data in samples between 150 to 450 participants (10, 11). Based on this benchmark and considering the annual number of stroke survivors referred to the TTSH inpatient stroke rehabilitation unit as well as a 20% rate of dropout or loss to follow-up, we targeted to enroll 400 participants in the study.

### 2.2 Data collection

At admission to the rehabilitation facility, the demographics, stroke characteristics, pertinent MRI or CT findings, important acute care data (Intensive Care Unit entry and duration), brain-related surgeries, acute hospital, and rehabilitation length of stay, and details about the prescribed rehabilitation program (therapy goals and content) will be obtained via TTSH electronic medical records. During the study, clinical, neurophysiological, and technology-assisted assessments as well as qualitative interviews will be performed to describe the multiple domains of post-stroke upper limb recovery (Table 1).

#### 2.2.1 Clinical assessment

##### 2.2.1.1 National Institute of Health Stroke Scale and Glasgow Coma Scale on admission

For ischemic stroke patients, the NIHSS will be used to objectively quantify the impairment caused by a stroke (30). The NIHSS (0–42) is composed of 11 items, each of which scores a specific ability between 0 and 4. For each item, a score of 0 typically indicates a normal function in that specific ability, while a higher score is indicative of some level of impairment. We will extract this admission data for ischemic strokes.

For hemorrhagic strokes, the GCS (total and subset eye opening, verbal and motor responses scores) which objectively describes the extent of impaired consciousness in all types of acute medical and trauma patients will be used (31). The scale assesses patients according to three aspects of responsiveness: eye-opening, motor, and verbal responses. Reporting each of these separately provides a clear, communicable picture of a patient. The findings in each component of the scale can aggregate into a total GCS which gives a less detailed description but can provide a useful summary of the overall severity. The GCS and its total score have since been incorporated into numerous clinical guidelines and scoring systems for victims of trauma or critical illness.

##### 2.2.1.2 Global disability level

We will measure the global disability level of our participants at T4, T5, and T6, corresponding to the time points of qualitative interviews. For this purpose, we will use the recommended clinical instrument—the modified Rankin scale (mRS) (32) for measurement. The mRS is a simple scale from 0–6 that measures the degree of disability or dependence in the daily activities of people who have suffered a stroke or other causes of neurological disability (see Table 2 for the scales of assessment tools).

**Table 2:** The scales of clinical assessment tools.

Description	Assessment tool	Scale
Global disability level	Modified Rankin Scale (mRS)	0–5
Upper limb motor impairments	Fugl-Meyer Assessment of the Upper Extremity (FMA-UE)	0–66
	Shoulder abduction and finger extension (SAFE) score	0–10
Upper limb activity capacity	Action Research Arm Test (ARAT)	0–57
Upper limb somatosensory impairments	Erasmus version of the Nottingham Sensory Assessment (NSA)	0–40
Upper limb spasticity	Modified Ashworth Scale (MAS)	0–4
Upper limb pain	Visual Analogue Scale (VAS)	0–10
Confidence in upper limb use	Upper Limb Self-Efficacy Test (UPSET)	0–200
Neglect	Bells Test	0–35
Trunk impairment	Trunk Impairment Scale (TIS)	0–23
Functional Independence	Functional Independence Measure (FIM)	18–126
Cognitive impairments	Montreal Cognitive Assessment (MoCA)	0–30
Quality of life	EQ-5D	Index score 0–1
		VAS score 0–100
	Stroke Specific Quality of Life (SSQOL)	49–245

##### 2.2.1.3 Upper limb motor impairments

Upper limb motor impairments will be measured with recommended clinical assessments (33, 34), including the Fugl–Meyer Assessment of the Upper Extremity (FMA-UE), shoulder abduction and finger extension (SAFE) scores, and grip strength.

The FMA-UE is a stroke-specific, performance-based impairment index (35). It is designed to assess motor functioning and coordination/speed in patients with post-stroke hemiplegia. It is applied clinically and in research to determine disease severity, describe motor recovery, and plan and assess treatment.

The SAFE score is commonly used in post-stroke recovery prediction models and is calculated by scoring shoulder abduction and finger extension separately, using the Medical Research Council grades, and summated to the SAFE score (36–38). The participant’s strength for each of these movements is scored between 0 and 5, where 0 is no muscle activity and 5 is normal strength and range of movement.

Grip strength is measured using a hand-held DynEx digital grip dynamometer (Fabrication Enterprises, Inc., NY, United States). The participant squeezes the dynamometer with all of their strength three times and an average score is calculated.

##### 2.2.1.4 Upper limb activity capacity

Upper limb activity capacity will be measured by the Action Research Arm Test (ARAT) (39), which evaluates upper limb motor capacity in a standardized format using 19 tests of motor function across 4 subsets: grasp., pinch, grip, and gross movement, both distally and proximally, following a stroke.

##### 2.2.1.5 Upper limb somatosensory impairment

Upper limb somatosensory impairment will be measured using the Erasmus version of the Nottingham Sensory Assessment (NSA) (40). The Erasmus NSA comprises assessments of tactile sensation (light pressure, pressure, and pinprick), sharp-blunt discrimination, two-point discrimination, and proprioception. For each item, a score of 0 denotes absent sensation, 1 denotes impaired sensation and 2 denotes normal sensation. The higher the score the better the preservation of sensation after stroke.

##### 2.2.1.6 Upper limb spasticity

We will describe the level of spasticity using the Modified Ashworth Scale (MAS) (41) for shoulder adductors, biceps, wrist flexors, and finger flexors. MAS measures resistance during passive soft-tissue stretching and is used as a simple measure of spasticity. The MAS is performed when moving the participant’s limb within their maximal range of motion within a second. It is scored from 0 to 4, with “0” indicating no increase in muscle tone while “4” indicating the affected part(s) is rigid in flexion and extension.

##### 2.2.1.7 Upper limb pain

We will describe the overall level of upper limb pain participants are experiencing using a visual analogue scale (VAS 0–10) (42). The VAS is a measurement instrument that tries to measure a characteristic or attitude that is believed to range across a continuum of values and cannot easily be directly measured. VAS pain is a unidimensional measure of pain intensity.

##### 2.2.1.8 Confidence in upper limb use

The participant’s confidence in upper limb use after stroke will be assessed using the Upper Limb Self-Efficacy Test (UPSET) (43). The UPSET is a questionnaire comprised of 20 questions to assess how confident the stroke survivors are in using their paretic upper limbs after stroke. For each question that represents a daily functional activity, the participant is asked to rate their level of confidence in using their paretic hand for that particular activity on a Likert scale from 0 to 10 (i.e., 0: not at all confident, 10: very confident).

##### 2.2.1.9 Neglect

The Bells Test will be used to assess the level of visual neglect in the participants with stroke (44). The participants will be asked to circle 35 black bells embedded among black distractors (i.e., pictures of houses and horses) on a page placed at the participant’s midline. The level of visual neglect is scored by how many bells have been omitted by the participant on a scoring sheet.

##### 2.2.1.10 Trunk impairment

The Trunk Impairment Scale (TIS) will be used to evaluate the trunk control of the participants (45). The TIS assesses static and dynamic sitting balance and trunk coordination in a sitting position, generating a total score between 0 and 23, where a higher score indicates better trunk control. Static sitting balance (score range 0–7) evaluates the ability to remain in a seated position with both feet on the floor and with the legs crossed. The dynamic sitting balance subscale (score range 0–10) assesses lateral flexion of the trunk, initiated from the upper and lower part of the trunk. The trunk coordination subscale (score range 0–6) assesses rotation from the shoulder and pelvic girdle in the horizontal plane.

##### 2.2.1.11 Functional independence in daily life

We will measure functional independence in daily life based on the Functional Independence Measure (FIM, score range 18–126) upon admission and discharge from the rehabilitation ward. The FIM includes measures of independence for self-care, including sphincter control, transfers, locomotion, communication, and social cognition (46). It is an 18-item, 7-level, ordinal scale intended to be sensitive to changes over the course of a comprehensive inpatient medical rehabilitation program.

##### 2.2.1.12 Cognitive impairments

Cognitive impairments will be rated by the Montreal Cognitive Assessment (MoCA) (47), which consists of simple tasks such as drawing, object naming, memory recall, reading, and mathematical operations (0, worst score, 30: best score).

##### 2.2.1.13 Quality of life

We will rely on EQ-5D and Stroke Specific Quality of Life (SSQOL) to measure the quality of life after stroke. EQ-5D is a generic quality-of-life measure and it includes mobility, self-care, usual activities, pain/discomfort, and anxiety/depression (48). The SSQOL measures 12 domains including mobility, energy, upper extremity function, work/productivity, mood, self-care, social & family roles, vision, language, thinking, and personality (49).

#### 2.2.2 Neurophysiological assessment (for participants with SAFE score <5 and without medical contraindications)

Transcranial magnetic stimulation (TMS) will be applied over the motor cortex to depolarize neurons and lead to motor evoked potentials (MEPs) for assessing the cortico-spinal excitability as well as resting motor threshold values (RMT) (50). MEPs are the electrical signals recorded from muscles following stimulation of motor pathways within the brain. Surface electromyography (EMG) electrodes are attached to the extensor carpi radialis (ECR) and first dorsal interosseous (FDI) for EMG recording. The “hot spot” of the MEP from ECR and FDI will be first identified in the contralesional (less affected) hemisphere, followed by the ipsilesional (more affected) hemisphere. TMS will be carried out using MagPro R30 (MagVenture, Inc., Denmark).

The main outcome measure of the TMS assessment is a patient’s MEP status, i.e., whether or not MEPs with consistent latencies can be evoked from the ipsilesional hemisphere.

Participants will be considered MEP positive if MEP of any amplitude but with consistent latency can be elicited while the ECR/FDI muscle is either at rest or is preactivated. If this criterion is not met even when stimuli are delivered at maximal intensity, the participant will be considered MEP negative.

For participants who are unable to achieve a RMT, an active motor threshold will be examined. The participant will perform an active wrist extension/make a fist with the less affected hand and hold it there during stimulation, while the investigator looks for a MEP in the ECR or FDI. For active motor threshold, the participant will be considered MEP positive if any amplitude of consistent latency can be elicited in 50% of at least 8 trials while the muscle is active.

Inspired by the PREP2 algorithm (36, 38), TMS measurements will only be formed at T0 and for patients with SAFE scores less than five. This may aid in the understanding of subgroups with good, limited, and poor recovery potential.

#### 2.2.3 Technology-assisted assessment

##### 2.2.3.1 Virtual Peg Insertion Test (VPIT)

**Figure 1:**
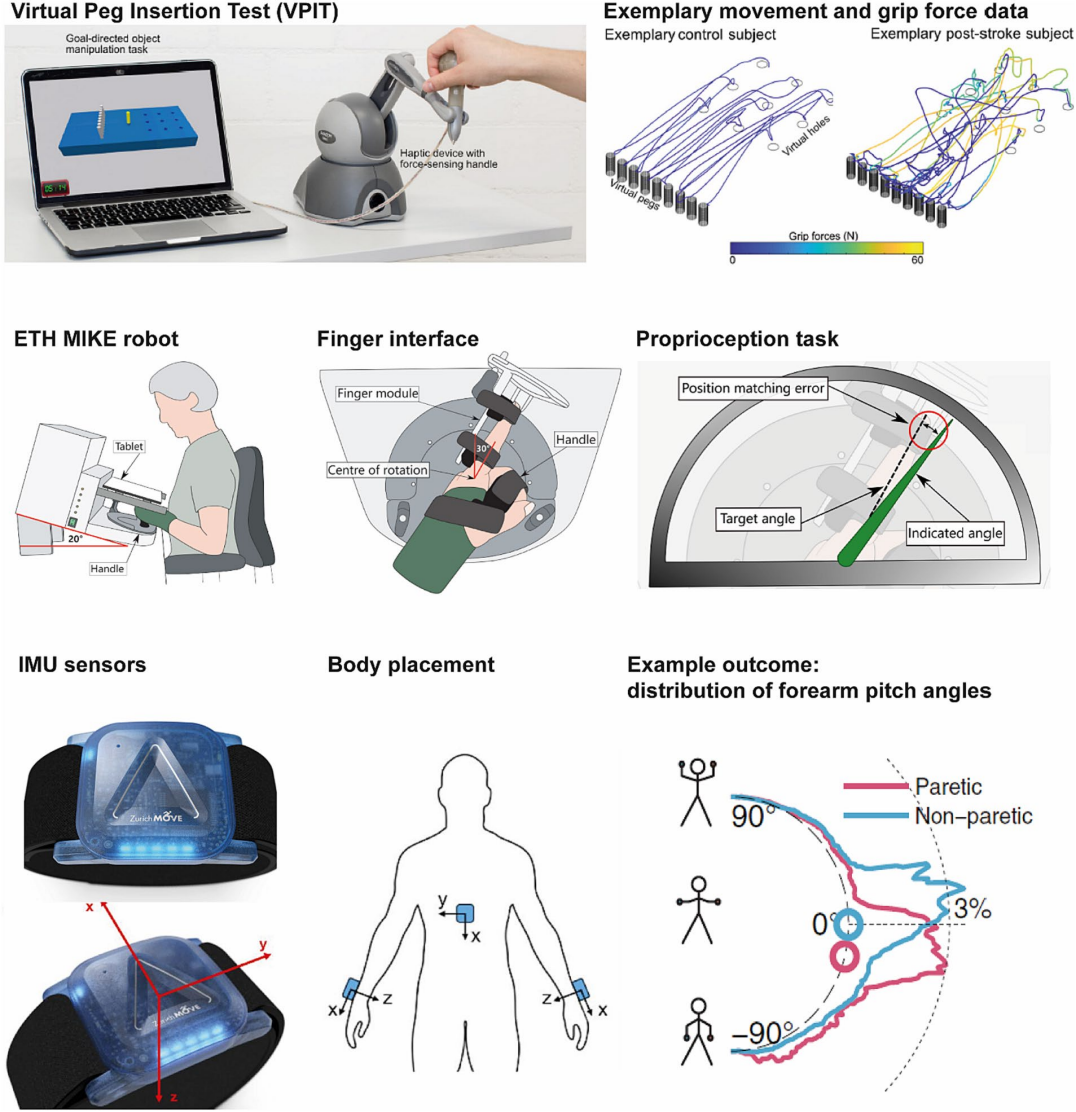
Technology-assisted assessments used in this study. Virtual Peg Insertion Test (VPIT, top), ETH MIKE (middle), and ZurichMove sensors (bottom).

The VPIT is a technology-assisted assessment that provides 10 sensitive and validated metrics describing upper limb movement patterns and hand grip forces (21, 22, 51, 52) (Figure 1). The VPIT consists of a virtual goal-directed pick-and-place task that requires coordinated arm and hand movements. It allows for gathering 3D upper limb endpoint kinematic and kinetic data in persons with mild to moderate upper limb impairments. The participant is instructed to squeeze and hold a commercial haptic device to pick up a peg and then insert it into one of the holes on the other side of the virtual board by releasing the force on the handle. The collected raw kinematic and kinetic data is transformed through a signal processing framework into 10 sensor-based digital biomarkers that provide information on different aspects of task performance, including for example movement smoothness, accuracy, and speed (21). These digital biomarkers and their clinimetric properties were previously validated in different neurological population (21, 22, 53). The data provide an objective, robust, and clinically feasible way to assess functionally relevant sensorimotor impairments in the arm and hand in chronic post-stroke individuals with mild to moderate sensorimotor deficits.

##### 2.2.3.2 ETH motor impairment and kinesthetic evaluation (MIKE)

The ETH MIKE is a one-degree-of-freedom end-effector device, that can be used to measure impairment of proprioception, motor and sensorimotor function in the metacarpophalangeal joint of the index finger (23, 54, 55). The ETH MIKE provides well-controlled stimuli to the index finger and sensitively measures the kinematic and kinetic responses, and has been shown to be safe and feasible in post-stroke individuals with mild to severe disability levels (23). Specifically, there is one task for proprioception assessment (gauge position matching (56)), with the absolute position matching error as the main outcome measure. Further, there are three tasks focused on motor impairments (active and passive range of motion, maximum force generation, and fast target reaching), providing as main outcomes active and passive range of motion, maximum finger force, and maximum movement speed for both flexion and extension. Additionally, one task is designed to evaluate the ability to integrate proprioceptive information during the execution of a complex movement, i.e., to assess sensorimotor impairments (trajectory following), providing the root means square tracking error as the main outcome measure. Thus, ETH MIKE is able to provide sensitive and objective measures of proprioception, motor impairments, and sensorimotor impairments that could inform the evolution of somatosensory and sensorimotor functions during recovery. Similar to the VPIT, the digital biomarkers used as outcome measures for the ETH MIKE were previously validated in different neurological population (23, 57, 58).

##### 2.2.3.3 ZurichMove

The ZurichMove sensors are custom-made wearable sensors developed by ETH Zurich (59) that allow unobtrusively monitoring the usage of the upper limb in the context of daily life activities.^1^ These sensors incorporate a 3-axis accelerometer, a 3-axis gyroscope and a 3-axis magnetometer. They can continuously record for approximately 3 days with a sampling frequency of 50 Hz. In this study, participants have to wear 5 ZurichMove sensors on both wrists, ankles and chest continuously for 48 h while performing all normal daily activities (in the clinic or at home for later time points). These sensors are attached to the body with non-gripping elastic bands by research staff or a caregiver and can be removed for showers and re-strapped by caregivers. Participants will record activities performed during the 48 h of wearing the sensors in a diary. Previously, the safety and usability of the wearable sensors have been established and the following outcome measures were defined and validated (60, 61). We aim to extract the duration of arms use in the clinical setting and during daily living by computing metrics such as upper limb activity counts as an approximation for the amount of limb usage (62), the gross movement score with an aim to identify functional relevant activities (60) and the GMAC (a fusion of both activity count and gross movement score) (63). The laterality ratio can be derived from these metrics to compare activity levels from both arms (64). These wearable sensors will provide digital biomarkers reflecting the usage of the more versus less affected upper limb in daily life.

#### 2.2.4 Psychosocial questionnaires and qualitative interviews

We will rely on repeated standardized questionnaires and qualitative interviews conducted in English, Mandarin, and Malay, in order to gain knowledge about the perspectives of individuals with stroke at 6, 12, and 24 months post-stroke. We will not conduct interviews at 3 months post-stroke as some stroke survivors may have not returned to the community in the Singaporean healthcare system. We also will not conduct interviews at 36 months post-stroke as the post-stroke experience may not have significantly changed after 2 years since stroke onset. The main aim is to explore stroke survivors’ experiences in the community qualitatively and quantitatively. Questions related to qualitative interviews focus on participants’ perception related to identity, impairments and the rehabilitation journey (see Supplementary Material). Additionally, we will delve into their perspectives on mental health, spirituality and social support with the following questionnaires: Beck Depression Inventory (65), Generalized Anxiety Disorder Scale (66), Modified Fatigue Impact Scale (67), Insomnia Severity Index (68), Social Support Survey (69), Life Balance Inventory (70) and Mindful Attention Awareness Scale (71). With regards to sample size calculation, guidelines recommended for qualitative samples have been relatively small, ranging from 20–40, to account for the complexity of the analysis. Given the longitudinal nature, the qualitative component of the study will include 30 participants or fewer, if data saturation is reached earlier, with sufficient cognitive function and willingness to share experiences coping with stroke. Interviews will be recorded and transcribed verbatim followed by thematic analyses.

#### 2.2.5 Data collection and management

Data from standardized clinical assessments will be collected by TTSH therapists and will be stored on the National Healthcare Group (NHG) REDcap platform electronically. Technology-assisted and neurophysiological outcomes will be collected by TTSH therapists and/or researchers from the Future Health Technologies (FHT) Programme and will be stored on the FHT data infrastructure SECure. Qualitative interviews will be conducted by researchers from the Rehabilitation Research Institute of Singapore, Nanyang Technological University (NTU).

Participants who withdraw from the study will not be replaced. All collected data will be used for analysis. We will also maintain contact with participants between time points to minimize the number of dropouts. In case of missing data, we will consider using controlled multiple imputation (72) to handle the missing data. Controlled multiple imputation is based on the assumption of missing at random and can be used as sensitivity analysis to evaluate the impact of missing data. Missing data will first be imputed following the Bayesian paradigm under the assumption of ignorability (i.e., missing at random). Each imputed dataset will then be analyzed using the substantive analysis model of interest. Finally, Rubin’s rules (73) are applied to give a single multiple imputation estimate for inference.

Regarding the data sharing plan, pseudonymized data will be shared in an access-controlled manner between research teams on a biweekly basis. Only anonymized or aggregated data will be shared with the general public such as researchers from other institutes or government agencies upon reasonable request.

#### 2.2.6 Statistical analysis

To understand post-stroke upper limb impairments and recovery, we will perform descriptive analyses for each outcome measure and various time points. Next, we will explore how different domains are interrelated and how the addition of digital biomarkers can inform key aspects of motor recovery and its dynamics using correlation matrices. We will investigate the relationship between movement quality (e.g., smoothness) and motor recovery, the relationship between somatosensory impairment and motor recovery, the relationship between upper limb use in activities of daily living and motor/somatosensory recovery, and whether these aspects present different time courses, or interact with each other.

To characterize upper limb recovery after stroke longitudinally, we will construct separate statistical models (e.g., mixed-effects models) that characterize the time course of upper limb impairments (FMA-UE) (3), activity capacity (ARAT) (4), activity performance (outcomes of wearable sensors), and quality of life (EQ-5D) post-stroke. We will then construct multivariate mixed models, leveraging on the uniqueness of the rich data set to probe the interaction and interdependence between various outcome measures and identify key determinants of recovery.

A mixture model based on a Bayesian approach (3), a clustering or subgroup identification analysis (74, 75) will then be used to identify subgroups of patients that exhibit distinct, clinically relevant recovery patterns based on a specific subset of variables identified in the above explorative analyses and their dynamics.

## 3 Discussion

To characterize longitudinal post-stroke upper limb recovery in sensorimotor function, functional independence, and quality of life in an Asian population, we designed a study protocol to gather rich information via clinical, technology-assisted, and neurophysiological assessments together with qualitative interviews at multiple time points from within 1 month until up to 3 years post-stroke. Our multimodal data set promises to deepen the understanding of the prevalence and relevance of specific upper limb impairments and their recovery trajectories on individual and group levels in stroke survivors. Based on these insights, refined clinical decision-making and tailored therapy programs could be established.

The uniqueness of our study includes (i) the use of technology-assisted assessments with multimodal assessments including clinical, neurophysiological, and qualitative assessments to understand post-stroke upper limb recovery, (ii) a relatively large Asian stroke population, and (iii) longitudinal follow-up for over 3 years. Clinical assessments are limited in their sensitivity due to ceiling effects and the use of ordinal scales (76). Questionnaires used to capture the usage of the upper limb in daily life have limited reliability and suffer from recollection bias (58). Here, we supplement clinical assessments with already validated assessment technologies including robotic devices, namely VPIT and ETH MIKE, as well as wearable sensors. The promise of digital biomarkers is that this approach will enable healthcare practitioners to objectively quantify recovery with substantially higher resolution, better sensitivity, and over a much longer period than currently available methods (77–79), adding credence to assessing real-world upper limb use from the acute to chronic stages of recovery. Kinematic-based measures may provide unique insights to describe movement quality and how this relates to upper limb impairment, functional ability and ultimately arm use in daily life. Furthermore, wearable sensor-based assessments can be done in the real-world natural environment during everyday activities, both in the hospital or at home, since they capture what stroke survivors actually do (performance) and not only what they could do (capacity) or wish to do (intention), as is the case in clinical assessments (60, 80, 81). Such applications in quantifying recovery over time are needed as they hold the potential to derive new outcome metrics, potentially supporting clinical decision-making and evaluating the efficacy of rehabilitation training.

However, previous post-stroke upper limb recovery studies were predominantly conducted in Caucasian populations with IS (14, 16, 17, 82). Asian stroke survivors have been found to have varied profiles compared to Caucasian populations (19–21), such as a high incidence rate of ICH, lacunar stroke, and young age potentially related to the high prevalence of uncontrolled hypertension and diabetes mellitus (26, 27). Given the difference between the Asian and Caucasian profiles, it is important to fill this knowledge gap by establishing a multimodal data set for monitoring upper limb recovery in Asian stroke survivors.

Additionally, studies that followed upper limb motor recovery post-stroke have found that recovery occurs at the greatest rate during the first 3 to 6 weeks post-stroke (14, 17, 82, 83). Although functional gains are largest during the first 3 months post-stroke and thereafter the recovery curve starts to flatten (16, 17, 82), recovery still occurs till 12 to 18 months post-stroke with some individuals experiencing recovery up to 24 months post-stroke (14) and possibly beyond. It is therefore important to observe and fully understand sensorimotor recovery longitudinally post-stroke in order to optimize neurorehabilitation outcomes even at a later stage after stroke. For these reasons, our study aims to establish longitudinal follow-ups for up to 3 years post-stroke to document how profiles of residual functions evolve, and whether interactions with Hr-QOL and self-efficacy may improve over a longer time frame. As such, the collected data could deepen our understanding of post-stroke upper limb recovery and potentially help reveal major determinants of recovery.

Importantly, our multimodal data set comprises variables that are highly relevant to post-stroke upper limb recovery, such as somatosensory function, self-efficacy, qualitative self-reported measures, Hr-QOL, MEP, and post-stroke experience. The somatosensory function is known to be of high importance in achieving full motor recovery, as recent studies highlighted that stroke survivors who had full motor recovery did not exhibit any impaired somatosensory function (84, 85). It suggests that somatosensory function could be an essential factor that may contribute to refining the resolution of prediction models, especially when it comes to fine functional ability. Nevertheless, there are limited research studies investigating post-stroke upper limb somatosensory recovery, primarily due to a lack of suitable clinical scales to reliably assess these. Upper limb somatosensory recovery, similar to motor recovery was shown to significantly occur 3–4 months post-stroke at the group level, while recovery continued to occur till 6 months post-stroke at the individual level (84). To our knowledge, studies looking into upper limb somatosensory recovery did not follow beyond 6 months post-stroke, hence the long-term trajectory of somatosensory recovery remains mostly unknown (84, 86, 87). These findings show that underlying interactions between motor and somatosensory recovery after stroke warrant further investigation to develop a greater understanding and insight into upper limb somatosensory recovery and its possible interactions with other modalities after stroke. The technology-assisted assessments included in this study will help collect a first data set to more objectively characterize somatosensory function and recovery.

Besides examining upper limb motor and somatosensory recovery after stroke, it is also important to investigate how these physical changes and recovery influence self-efficacy in upper limb use, qualitative self-reported outcomes, and Hr-QOL after stroke. To date, there is still a dearth of information in this area. It has been reported that chronic stroke survivors expressed the loss of their upper limb function as an “enormous loss” (88). It can be seen that physical impairments may possibly give rise to other related problems in self-efficacy, qualitative self-reported measures, and Hr-QOL. Thus, it is important to longitudinally track these parameters beyond 1 year to understand how physical upper limb recovery impacts other aspects of stroke survivors’ lives. Complemented with qualitative interviews, information on post-stroke experiences such as coping strategies and difficulties in daily life could be obtained. It allows researchers to construct a holistic viewpoint of stroke consequences in stroke survivors and gain knowledge about the associations between functional recovery, daily activities, participation, environmental factors, and personal factors. Hence it is the aim of our study to establish a comprehensive database of not only physical recovery but also other factors such as self-efficacy, qualitative self-reported measures, and Hr-QOL to yield information on important factors influencing upper limb sensorimotor recovery trajectory across time. Nevertheless, gathering self-reported outcomes would require sufficient cognitive function from the responders and this might limit the generalizability of our findings.

Additionally, evaluating the integrity of the corticospinal tract with TMS has been used for predicting the upper limb recovery potential of stroke patients as formalized by the PREP2 algorithm (38). However, there has been some debate as to whether MEP measures improve prediction over and above the information provided by clinical tests (89–91), particularly, when the MEP status cannot be tested within the first 7 days post-stroke (37, 38). The current center’s acute stroke unit and rehabilitation unit are in separate physical locations with differential access to TMS machines, hence the predictive power of an early MEP status is limited (36). We will investigate whether MEP-negative stroke survivors have a bad prognosis for recovering upper limb function and whether this information sheds light on the recovery trajectory of individual patients with poor function early after stroke and whether obtaining MEP status would benefit clinical decision-making.

In conclusion, this study aims to develop and implement a technology-assisted digital biomarker (TAILOR) platform for describing upper limb sensorimotor recovery in Asian stroke survivors. Digital biomarkers could reduce measurement errors and improve the accuracy of outcome measures to complement clinical, neurophysiological, and qualitative assessments and could further offer therapeutic and prognostic value in stroke neurorehabilitation. With a gathered multimodal database, it is advantageous to characterize the pattern of upper limb recovery and to identify subgroups of stroke survivors that exhibit distinct, clinically relevant recovery patterns. This data set can consequently be used in the future to establish prediction models of upper limb recovery in stroke survivors receiving conventional rehabilitation training. This will aid clinical decision-making, therapeutic planning, and the development of tailored interventions.

## Ethics statement

The studies involving human participants were reviewed and approved by ETH Zurich’s Ethics Commission (EK 2021-N-181) in Switzerland and the National Healthcare Group’s Domain Specific Review Board (NHG DSRB Ref: 2021/00919) in Singapore. The participants will provide their written informed consent to participate in this study.

## Author contributions

H-JC, LFC, and CMK were involved in the general study design and contribution to the whole manuscript draft. LFC, RL, CWKK, SK, SKW, EJ, TS, AS, PCG, EL, MZ-M, PL, and KC contributed to the design of the assessment protocol. RG, OL, and NW contributed to the study conception and design. All authors contributed to the article and approved the submitted version.

## Footnotes

^1^ www.zurichmove.com
