# Supplementary material for: Upper limb sensorimotor recovery in Asian stroke survivors: a study protocol for the development and implementation of a Technology-Assisted dIgitaL biOmaRker (TAILOR) platform
Source: Front Neurol. 2023 Nov 30;14:1246888. doi: 10.3389/fneur.2023.1246888 (PMC10722087; doi:10.3389/fneur.2023.1246888)
Supplement: Supplementary file 1 [file Data_Sheet_1.docx]

Supplementary Material

Upper Limb Sensorimotor Recovery in Asian Stroke Survivors: A Study Protocol for the Development and Implementation of a Technology-Assisted dIgitaL biOmaRker (TAILOR) Platform

Hsiao-ju Cheng^†^, Lay Fong Chin^†^, Christoph M. Kanzler, Rea Lehner, Christopher W. K. Kuah, Simone Kager, Eva Josse, Tengiz Samkharadze, Ananda Sidarta, Pablo Cruz Gonzalez, Eloise Lie, Monika Zbytniewska-Mégret, Seng Kwee Wee, Phyllis Liang, Roger Gassert, Karen Chua^‡^, Olivier Lambercy^‡^, Nicole Wenderoth^‡,^*

*** Correspondence:** Prof. Nicole Wenderoth: [nicole.wenderoth@hest.ethz.ch](mailto:nicole.wenderoth@hest.ethz.ch)

†These authors share the first authorship.

‡These authors share the last authorship.

# Qualitative Interview Questions

## T4: 6 months post-stroke

- Could you please describe what a typical day was like for you before the stroke?
- Could you share more about your work and the roles you had, as well as your family life and hobbies before the stroke?
- On the day you had the stroke, can you recall what you were doing and the events leading up to it?
- Can you describe the events before and after the stroke, especially within the first 24 hours?
- Can you describe the impairments you have experienced since the stroke and how they impact your daily life?
- Can you share what a typical day looks like for you now?
- Could you please share details about the rehabilitation program and therapies you have been undergoing since the stroke?
- Can you share a specific good experience you had during rehabilitation that helped your recovery?
- Do you recall a negative experience during rehabilitation that was especially notable to you?
- Could you share whether you feel motivated or unmotivated to attend the rehabilitation sessions?
- As compared to before the stroke, have you noticed any significant changes in your mental health over time?
- How have your family and friends been involved in supporting you throughout your rehabilitation and recovery journey?

## T5: 1 year post-stroke

- Could you describe the ongoing impairments, sequels you are experiencing after the stroke, and how these impairments have evolved over time?
- Could you describe what a typical day is like for you now?
- What types of therapy have you been receiving, and how frequently?
- How do you perceive the impact of your emotional health on your rehabilitation process?
- Have you noticed if the rehabilitation process has influenced your emotional health?
- Could you share whether you feel motivated or unmotivated to attend the rehabilitation sessions?
- Are there specific goals you are aiming to achieve, or particular areas of improvement you are focusing on?
- Have you ever wondered what your life would be like if you could recover faster and better?
- What improvements do you think could be made to the healthcare support available to stroke survivors?
- What aspects do you believe are currently working well in the healthcare rehabilitation system?
- Can you share your experience on how stroke has influenced on your spiritual beliefs and practice?
- Have you experienced feelings of loneliness since surviving the stroke?
- In your interactions with the broader community, have you noticed any changes in how people perceive or interact with you since the stroke?
- How has your communication with family and friends about your needs and expectations changed since your stroke?

## T6: 2 years post-stroke

- What activities, routines, or aspects of your life do you remember most before the stroke?
- What do you find yourself missing the most about your life before the stroke?
- How has your daily routine changed compared to before the stroke?
- Can you discuss the types of therapy you are currently receiving?
- What have been the most significant milestones in your journey of recovery?
- What are your expectations regarding your recovery in the long term?
- When comparing your current perspective with the early stages after the stroke and the year, how have your thoughts and aspirations transformed or stayed the same?
- Have your spiritual beliefs and the experience of the stroke influenced your thoughts on mortality and the afterlife?
- Since having stroke, how has your social network changed or developed?
- Have you ever experienced discrimination from others in your community?
- Can you describe specific moments or activities that bring you happiness now?
- Have you ever wondered what your life would be like if you had not had the stroke?
- Is there anything specific that has provided you comfort and peace during your recovery?
- How has the stroke influenced your perspective on life and the world around you?
